# Data assimilation of ambient concentrations of multiple air pollutants using an emission-concentration response modeling framework

**DOI:** 10.3390/atmos11121289

**Published:** 2020

**Authors:** Jia Xing, Siwei Li, Dian Ding, James T. Kelly, Shuxiao Wang, Carey Jang, Yun Zhu, Jiming Hao

**Affiliations:** 1 State Key Joint Laboratory of Environmental Simulation and Pollution Control, School of Environment, Tsinghua University, Beijing 100084, China; 2 State Environmental Protection Key Laboratory of Sources and Control of Air Pollution Complex, Beijing 100084, China; 3 School of Remote Sensing and Information Engineering, Wuhan University, Wuhan 430079, China; 4 State Key Laboratory of Information Engineering in Surveying, Mapping and Remote Sensing, Wuhan University, Wuhan 430079, China; 5 Office of Air Quality Planning and Standards, U.S. Environmental Protection Agency, Research Triangle Park, NC 27711, USA; 6 College of Environment and Energy, South China University of Technology, Guangzhou Higher Education Mega Center, Guangzhou 510006, China

**Keywords:** data assimilation, response model, ozone, PM_2.5_, emission inversion

## Abstract

Data assimilation for multiple air pollutant concentrations has become an important need for modeling air quality attainment, human exposure and related health impacts, especially in China that experiences both PM_2.5_ and O_3_ pollution. Traditional data assimilation or fusion methods are mainly focused on individual pollutants, and thus cannot support simultaneous assimilation for both PM_2.5_ and O_3_. To fill the gap, this study proposed a novel multipollutant assimilation method by using an emission-concentration response model (noted as RSM-assimilation). The new method was successfully applied to assimilate precursors for PM_2.5_ and O_3_ in the 28 cities of the North China Plain (NCP). By adjusting emissions of five pollutants (i.e., NO_x_, SO_2_, NH_3_, VOC and primary PM_2.5_) in the 28 cities through RSM-assimilation, the RMSEs (root mean square errors) of O_3_ and PM_2.5_ were reduced by about 35% and 58% from the original simulations. The RSM-assimilation results small sensitivity to the number of observation sites due to the use of prior knowledge of the spatial distribution of emissions; however, the ability to assimilate concentrations at the edge of the control region is limited. The emission ratios of five pollutants were simultaneously adjusted during the RSM-assimilation, indicating that the emission inventory may underestimate NO_2_ in January, April and October, and SO_2_ in April, but overestimate NH_3_ in April and VOC in January and October. Primary PM_2.5_ emissions are also significantly underestimated, particularly in April (dust season in NCP). Future work should focus on expanding the control area and including NH_3_ observations to improve the RSM-assimilation performance and emission inventories.

## 1. Introduction

Human exposure to air pollutants such as ozone (O_3_) and fine particulate matter (PM_2.5_) has been associated with considerable adverse health effects. In 2017, 2.9 million premature mortalities were attributed to PM_2.5_ exposure globally and about a half-million mortalities were attributed to O_3_ exposure [1–2]. Accurate estimation of air pollutant concentrations and their exposures is critical for assessing health impacts and developing emission control strategies. Previous studies have demonstrated that the assimilation of chemical transport model (CTM) simulations with monitor observations can provide spatiotemporally continuous estimates of air pollutant concentrations and corresponding exposure while incorporating the accuracy of in-situ monitoring data and the spatiotemporal continuity of CTM modeling. Traditional data fusion methods such as Voronoi Neighbor Averaging (VNA) [3], enhanced Voronoi Neighbor Averaging (eVNA) [4], and Downscaler (DS) [5–6] have been applied in many studies to estimate air pollutant concentrations, human exposure and related health impacts [7]. Concentration estimates based on fusing monitor and CTM data can be helpful in characterizing the effects of control strategies for air quality attainment [8].

However, traditional data fusion is mostly based on statistical interpolation or regression methods that are designed to predict each pollutant individually. The need for simultaneous assimilation of multiple pollutants is evident with the deterioration of O_3_ pollution in China corresponding to PM_2.5_ improvements [9]. The challenge for multipollutant assimilation is to wisely design certain constraints to maintain the natural linkages between pollutants during the assimilation process. Specifically, the natural linkage between O_3_ and PM_2.5_ is that both pollutants have contributions from common precursors (NO_x_ and VOC), similar atmospheric diffusion/advection transport, and chemical oxidation reactions. Therefore the modulation of one pollutant concentration should exert corresponding influence on the other, and these connections should be represented during the assimilation. Traditional data fusion methods like eVNA can only fuse pollutant concentrations separately without considering multipollutant linkages. However, assimilating O_3_ and PM_2.5_ separately will result in different NO_x_ and VOC adjustment ratios since the optimization is conducted individually for one pollutant instead of both. The simultaneous assimilation for both pollutants can result in a consistent adjustment of NO_x_ and VOC emissions and also provide additional constraints to ensure the inversion problem is well posed [10]. Therefore, the development of an advanced data assimilation method is necessary to support simultaneous data fusion for multiple pollutants to accurately represent current air quality and the effectiveness of future control policies.

Discrepancies between predictions and observations are associated with uncertainties in many factors such as model emissions, resolution, chemical mechanisms, and process parameterizations as well as measurement errors. Among these factors, uncertainties in emission inventories are regarded as one of the largest contributors to the biases in CTM predictions. Unfortunately, the interpolation-based fusion methods like eVNA do not provide information on the contributors to model errors. Inversion modeling studies have used advanced assimilation techniques such as ensemble Kalman filtering to correct the emissions simultaneously during the assimilation process [11]. The revised emissions are also useful for improving emission inventories [12–13]. Ideally, the predictions developed by combining CTM simulations and observations would provide not only accurate and spatiotemporally continuous concentrations of multiple pollutants, but also corrections to emission inventories, which are one of the largest contributors to model biases. To date, however, most inversion studies only focused on individual pollutants that can be measured directly. Therefore, there remains a lack of inverse emissions estimates for multiple pollutants including those that cannot be directly observed, such as primary fine particulate matter (noted as pPM_2.5_).

The recently developed response surface model (RSM) provides real-time prediction of both PM_2.5_ and O_3_ using emission-concentration relationships. The RSM can identify the emission control factors needed to meet air quality targets, and thus provides information on the changes in emissions of multiple pollutants needed to improve air quality predictions against monitoring data [14–15]. Advanced machine learning techniques enables its fast application across any time period and spatial location [16]. Different from inversion modeling, the RSM modifies anthropogenic emissions of five pollutants at the regionally aggregated level (by city in this study) based on an assumption that the spatial distribution of emissions is relatively accurate compared with emission magnitudes. In combination with surface observations from a few monitoring sites, the RSM has been successfully applied to investigate the emission changes during the COVID-19 period in North China Plain (NCP) [17]. The RSM-based assimilation can well maintain the inner linkages of PM_2.5_ and O_3_, as the RSM prediction can be considered as one CTM simulation under a specific emission scenario. The emission adjustment ratios from the RSM-assimilation also address the question of how emissions should be modified to achieve a certain level of agreement between predictions and observations.

In this study, an emission-concentration response modeling framework is established based on the RSM (noted as RSM-assimilation). Then its performance in the data assimilation of ambient concentrations of multiple air pollutants is tested in the case study of NCP. The new RSM-assimilation method is described in Section 2. The performance of the RSM-assimilation approach as well as the implications for improving the emission inventory is discussed in Section 3. Advantages, limitations and future work on RSM-assimilation are summarized in Section 4.

## 2. Methods

### 2.1. Simulation and observation data

The principle of RSM-assimilation is to nudge the CTM simulation toward available observations by adjusting the emissions. The CTM used in this study is the Community Multiscale Air Quality (CMAQ, version 5.2.1, www.epa.gov/cmaq) model, and the meteorological fields are based on a simulation with the Weather Research and Forecasting (WRF, version 3.8) model. The same configuration of WRF-CMAQ was applied as in our previous studies [18]. The Morrison double-moment microphysics scheme [19], the Rapid Radiative Transfer Model [20], Kain-Fritsch cumulus cloud parameterization [21], Pleim-Xiu land-surface physics scheme [22], and the Asymmetric Convective Model for the planetary boundary layer (PBL) physics scheme [23] were used in WRF simulation. We used the Carbon Bond 6 [24] and the AERO6 aerosol module [25] for gas-phase and particulate matter chemical mechanisms, respectively.

**Figure 1.**
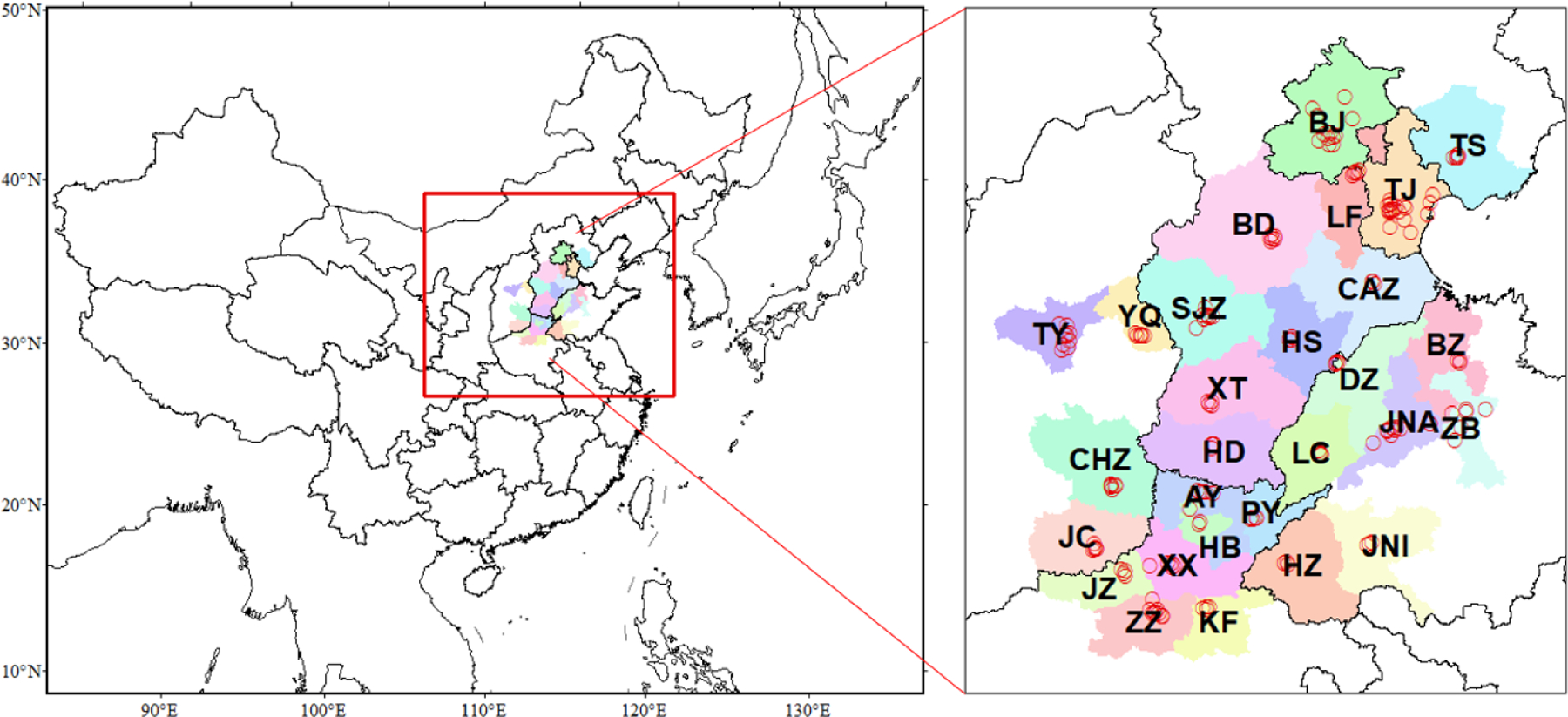
Simulation domain and observation sites in 2+26 cities of North China Plain (red dots: surface monitor sites for NO_2_, SO_2_, O_3_ and PM_2.5_; the 28 cities are BJ-Beijing, TJ-Tianjin, BD-Baoding, CAZ-Cangzhou, HD-Handan, HS-Hengshui, LF-Langfang, SJZ-Shijiazhuang, TS-Tangshan, XT-Xingtai, TY-Taiyuan, YQ-Yangquan, ZZ-Zhengzhou, JZ-Jiaozuo, AY-Anyang, HB-Hebi, XX-Xinxiang, KF-Kaifeng, PY-Puyang, HZ-Heze, LC-Liaocheng, DZ-Dezhou, JNI-Jining, ZB-Zibo, JNA-Jinan, BZ-Binzhou, JC-Jincheng, CHZ-Changzhi).

The simulation domain covers the 28 key cities in NCP, as shown in Figure 1. The simulations of the 27 km × 27km China domain provide the boundary conditions for the simulation of the nested domain of 9km × 9km over NCP. The anthropogenic emissions of 28 cities are based on the Multi-resolution Emission Inventory for China (MEIC) dataset for the year of 2016 [26], because the data for 2017 was not available when we initiated this work. The emissions include five major sectors, i.e., industry, power, residential, transportation, and agriculture. The gridded emissions of five air pollutants over 28 cities in NCP are shown in Figure S1. Biogenic emissions were generated by the Model for Emissions of Gases and Aerosols from Nature (MEGAN) version 2.0 [27]. The inline dust model was used to estimate the wind-blown dust emissions in the 27km × 27km China domain. Considering the wind-blown dust mostly comes from outside of NCP, we turned off the inline dust model for the simulation of 9km × 9km over NCP to reduce the computational burden associated with the RSM model development. The performance of the WRF-CMAQ model in simulating meteorological variables and pollutant concentrations was evaluated by comparing with surface observations [28]. The WRF-CMAQ model well reproduces the observed meteorology, with mean biases within ±0.5° for 2-meter temperature, ±1g/kg for 2-meter humidity, ±0.5m/s for 10-meter wind speed, and 10° for wind direction. The model also exhibits acceptable performance in simulating PM_2.5_ and O_3_ concentrations, with slight low-biases in PM_2.5_ and high-biases in O_3_. Such biases can be reduced through the data assimilation with RSM, as discussed in the Section 3.1.

The RSM model was developed based on 21 emission-control scenario simulations with the WRF-CMAQ model by implementing polynomial functions to represent the response of O_3_ and PM_2.5_ to emission changes of five pollutants including SO_2_, NOx, VOC, NH_3_ and pPM_2.5_ in 28 cities in NCP [14–15]. The polynomial functions of O_3_ and PM_2.5_ response to emissions are shown as E1–2.

(E1) $$\Delta PM_{2.5}=a_{1}E_{NOx}^{4}+a_{2}E_{NOx}^{3}+a_{3}E_{NOx}^{2}+a_{4}E_{NOx}+a_{5}E_{NOx}E_{NH3}+a_{6}E_{NOx}^{4}E_{VOCs}+a_{7}E_{NOx}^{2}E_{VOCs}+\;a_{8}E_{NOx}E_{VOCs}+a_{9}E_{SO2}+a_{10}E_{NH3}^{3}+a_{11}E_{NH3}^{2}+a_{12}E_{NH3}+a_{13}E_{VOCs}^{2}+a_{14}E_{VOCs}+a_{15}E_{pPM2.5}$$

(E2) $$\Delta O_{3}=a_{1}E_{NOx}^{5}+a_{2}E_{NOx}^{4}+a_{3}E_{NOx}^{3}+a_{4}E_{NOx}^{2}+a_{5}E_{NOx}+a_{6}E_{NOx}^{5}E_{VOCs}+a_{7}E_{NOx}^{2}E_{VOCs}+\;a_{8}E_{NOx}E_{VOCs}+a_{9}E_{NOx}E_{VOCs}^{3}+a_{10}E_{SO2}+a_{11}E_{NH3}+a_{12}E_{VOCs}+a_{13}E_{VOCs}^{2}+a_{14}E_{VOCs}^{3}+a_{15}E_{pPM2.5}$$

where $\Delta O_{3}$ and $\Delta PM_{2.5}$ is the response of O_3_ and PM_2.5_ concentrations (i.e., change to the baseline concentration), respectively, at each simulated grid cell; *E*_*NOx*_, *E*_*SO2*_, *E*_*NH3*_, *E*_*VOCs*_, and *E*_*pPM2.5*_ is the change ratio of NO_x_, SO_2_, NH_3_, VOCs, and pPM_2.5_ emissions, respectively, relative to baseline (i.e., baseline = 0); $a_{i}$ is the coefficient of term *i* which is determined by fitting with 21 emission-control scenario simulations.

The RSM can predict O_3_ and PM_2.5_ responses to emission changes in good agreement with CMAQ predictions, as the out-of-sample validation of RSM predictions against CMAQ simulations yields NMBs (Normalized Mean Biases) within ±1% [28].

We gathered the observed NO_2_, SO_2_, O_3_ and PM_2.5_ measurements from monitoring sites in the 28 NCP cities from the China National Environmental Monitoring Centre (http://www.cnemc.cn/en/) (Figure 1). The daily maximum 8-hour O_3_ and daily averaged PM_2.5_ concentrations were assimilated for the modeling domain with 9-km horizontal grid spacing. The simulation period is January, April, July and October in 2017 to represent winter, spring, summer and fall, respectively. Assimilation performance was evaluated using the Root Mean Square Error (RMSE), Normalized Mean Bias (NMB) and R-squared (R^2^).

In the baseline case of RSM-assimilation, we included all observation sites during the assimilation. To compare with the simulation, we averaged observations from sites within the same model grid cell (9km ×9km). This resulted in about 85 sites (the number is smaller than that shown in Figure 1) across 28 cities to be used in data assimilation. The monitoring sites are mostly located at the urban center of each city, were both local emission sources and regional contributions influence the ambient PM_2.5_ and O_3_ concentrations [29–30]. The RSM-assimilation can account for both local formation and regional transport since it was original built from CMAQ model simulations. Details about the numbers of sites used for nudging in each city are summarized in Table S1. Additionally, we designed single-site cases for each of the 28 cities in which only a single randomly-selected site was assimilated for each city. These cases were used to examine the sensitivity of the RSM-assimilation method to the number of observation sites.

### 2.2. RSM-assimilation framework

**Figure 2.**
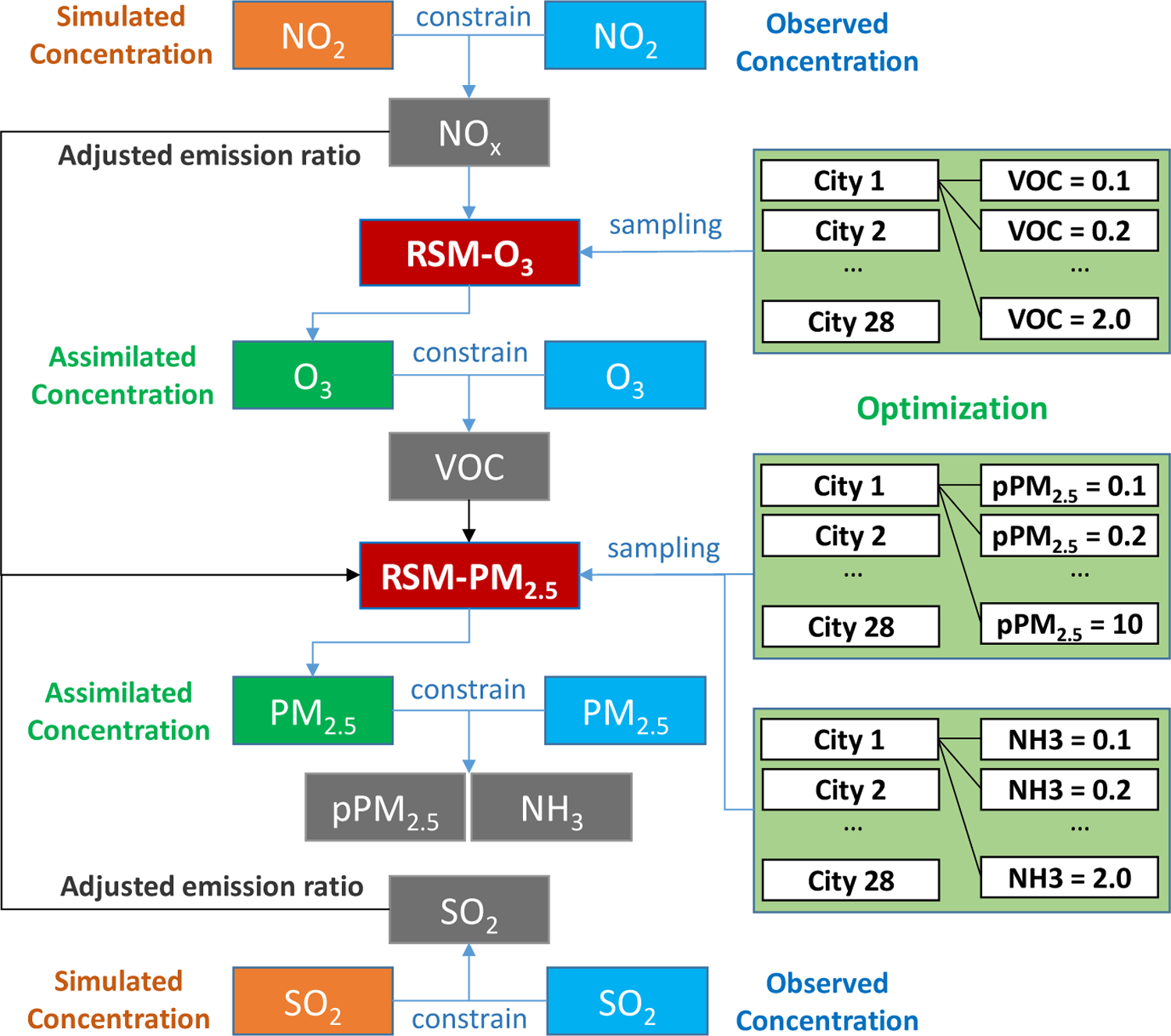
The emission-concentration-based response modeling framework. (In assimilating observational data, NO_x_ and SO_2_ emissions are adjusted first, followed by optimization for VOC and then pPM_2.5_ and NH_3_ emissions)

Figure 2 presents the framework for the newly developed RSM-assimilation method. As in our previous study [17], we first estimate the adjusted emission ratios of NO_x_ and SO_2_ based on the comparison of simulated and observed NO_2_ and SO_2_ concentrations. Next, an optimization process is implemented that samples VOC emission ratios as inputs for the RSM-O_3_ model. This process results in optimized VOC emission ratios for the 28 cities that yield the best agreement between simulated and observed O_3_ concentrations at the monitoring sites (determined by the average RMSE over all sites). A similar optimization process is then applied for PM_2.5_, where the adjusted NO_x_, SO_2_ and VOC emission ratios are introduced into the RSM-PM_2.5_ model and emission ratios are sampled for NH_3_ and pPM_2.5_ in the 28 cities. Since direct observations of surface NH_3_ concentrations are unavailable, the optimized pPM_2.5_ and NH_3_ emission ratios correspond to the assimilated PM_2.5_ concentrations that agree most closely with observed PM_2.5_ concentrations among all possible combinations of emission ratios. The optimization was conducted through a multiple looping process to select the best combination of emission ratios (with a small step of 0.05) for different pollutants and cities to achieve the best overall agreement. As currently designed, the RSM is suitable for emission ratios of gas pollutants that range from 0 (fully controlled emissions) to 2 (doubled emissions). The response functions of O_3_ and PM_2.5_ to emission changes of gaseous precursors were fitted through the regression of multiple emission-control scenario simulations (emission ratios from 0–2); thus, the uncertainties of the response functions will be considerably larger when the emissions vary outside of the 0–2 emission ratio range. Here, we limit the adjusted emission ratios of NO_2_, SO_2_, VOC, and NH_3_ to range from 0.1 (90% reduction) to 2.0 (100% increase). We allow a wider range for pPM_2.5_ emission ratios due to the large uncertainties in pPM_2.5_ emissions, particularly for fugitive emission sources like wind-blown dust. Unlike the gas pollutants, the RSM has no limits on the adjusted emission ratios for pPM_2.5_, since impacts of these emissions are represented linearly in the RSM [15].

## 3. Results

### 3.1. Performance of RSM-assimilation

**Figure 3.**
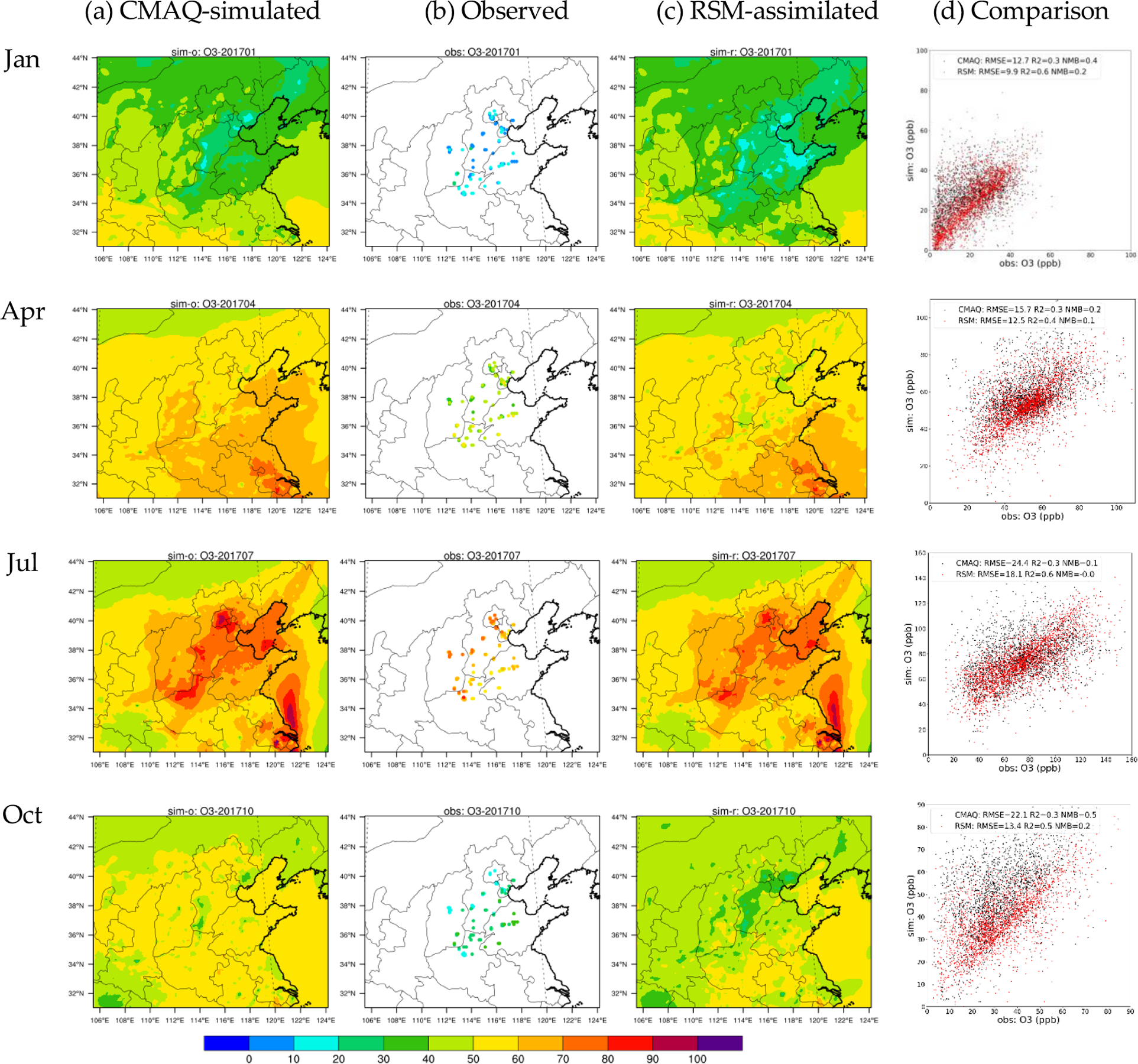
Comparions of CMAQ-simulated, observed, and RSM-assimilated O_3_ concentrations

**Figure 4.**
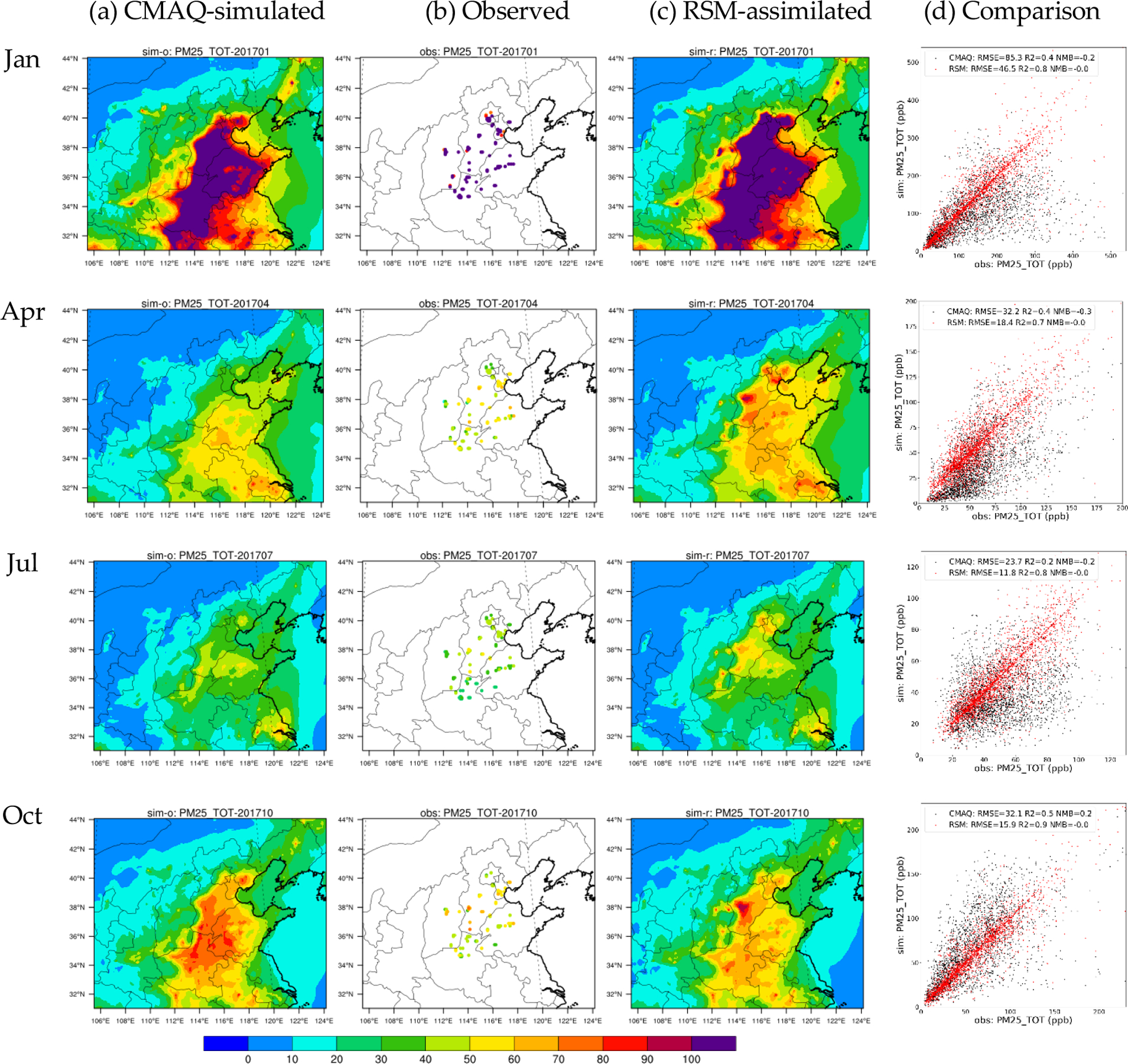
Comparions of CMAQ-simulated, observed, and RSM-assimilated PM_2.5_ concentrations

In Figure 3 and 4, the assimilated O_3_ and PM_2.5_ concentrations in the baseline RSM-assimilation case are compared with the original CMAQ simulations in terms of the spatial pattern of monthly averaged concentrations and with observations in terms of daily paired values for sites in the 28 cities. The assimilated spatial fields generally maintain the spatial distribution of the original CMAQ simulation, but have slightly modulated concentrations in areas surrounding the observation sites that better reflect observed values. The accuracy of both O_3_ and PM_2.5_ concentrations is enhanced through the RSM-assimilation that reduces the RMSE from 12.7–24.4 ppb (pre-assimilation) to 9.9–18.1 ppb (post-assimilation) for O_3_, and from 23.7–85.3 μg m^−3^ (pre-assimilation) to 11.8–46.5 μg m^−3^ (post-assimilation) for PM_2.5_.

The high biases in simulated O_3_ are greatly reduced through RSM-assimilation, as the NMBs decrease from up to 50% in CMAQ to within 20% in RSM-assimilation. The underestimation of PM_2.5_ is also mitigated through RSM-assimilation, as the NMBs for PM_2.5_ are reduced to 0%. The comparison of daily paired predictions and observations indicates large improvements for R^2^ in RSM-assimilation: e.g., the R^2^ for PM_2.5_ predictions increased from 0.2–0.5 (pre-assimilation) to 0.7–0.9 (post-assimilation). However, the RSM-assimilation is much more effective for PM_2.5_ than for O_3_. This behavior might be associated with the large contributions to O_3_ from sources that cannot be adjusted through RSM assimilation (e.g., biogenic sources) and the effectiveness of primary PM_2.5_ emissions for modulating PM_2.5_ concentrations. We note that the observations displayed in Figure 4 are distributed in discrete locations across the domain, and do not necessarily match the location of the simulated and assimilated concentrations (see Figure S2 for the matched location comparisons).

Time-series comparisons for O_3_ and PM_2.5_ in the 28 cities (Figure S3 and S4, respectively) suggest similar levels of improvement, as the RMSE was reduced after the four-month assimilation across the 28 cities.

**Figure 5.**
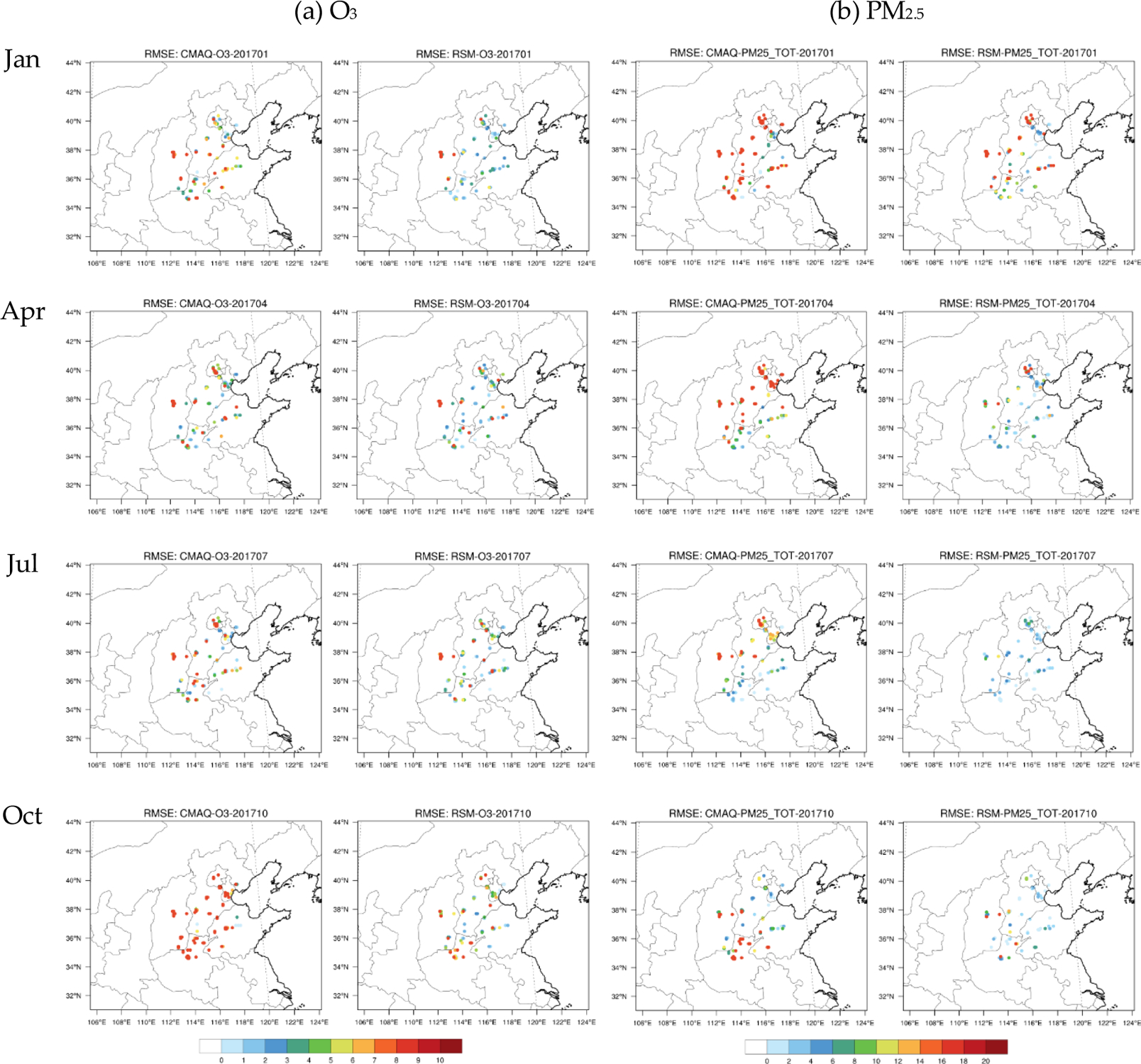
Comparisons of RMSE in CMAQ-simulated and RSM-assimilated concentrations of O_3_ (ppb) and PM_2.5_ (μg m^−3^) across all observation sites

Although the RSM-assimilation improves the simulation accuracy, large discrepancies in the performance improvements are evident among the 28 cities. To further investigate the variation of RSM-assimilation performance across all observation sites, we compared the RMSEs of CMAQ-simulated and RSM-assimilated O_3_ and PM_2.5_ in the 28 cities at the four-month averaged level, as shown in Figure 5.

In general, the RSM-assimilation is less effective in reducing the RMSE for sites in cities at the edge of the control region (i.e., the full 28-city area). The smallest O_3_ improvements occurred in cities such as ZB on the eastern edge of the control region and YQ on the western edge of the control region. These cities had relatively large RMSEs after the assimilation, with RMSE reductions of only 6% (ZB) and −3% (YQ) (slightly worse performance) relative to the CMAQ simulation. For the other cities, the O_3_ improvements are much greater, with RMSE reductions of at least 16%. For PM_2.5_, YQ also has the smallest improvement, with a 24% reduction in RMSE (compared to a 50–80% for the other cities). Such results indicate that the RSM-assimilation has limited ability to improve the accuracy of concentrations at the edge of the control region, where the influence of emissions from outside of the control region is large. Enlarging the control area or combining with an RSM model based on the larger domain is recommended to improve the ability of RSM-assimilation for those cities. Meanwhile, discrepancies also exist within a city—e.g., RMSE was reduced in eastern Tianjin but increased in western Tianjin in Jan. This behavior occurs because the RSM-assimilation adjusts emissions at the city averaged level and maintains the spatial distribution of emissions within each city at the a priori estimate. Future improvement of the spatial distribution of the emission within the city is also recommended by adopting additional observations like satellites and advanced technologies like machine-learning to address such limits.

### 3.2 Sensitivity of RSM-assimilation to the site number

**Figure 6.**
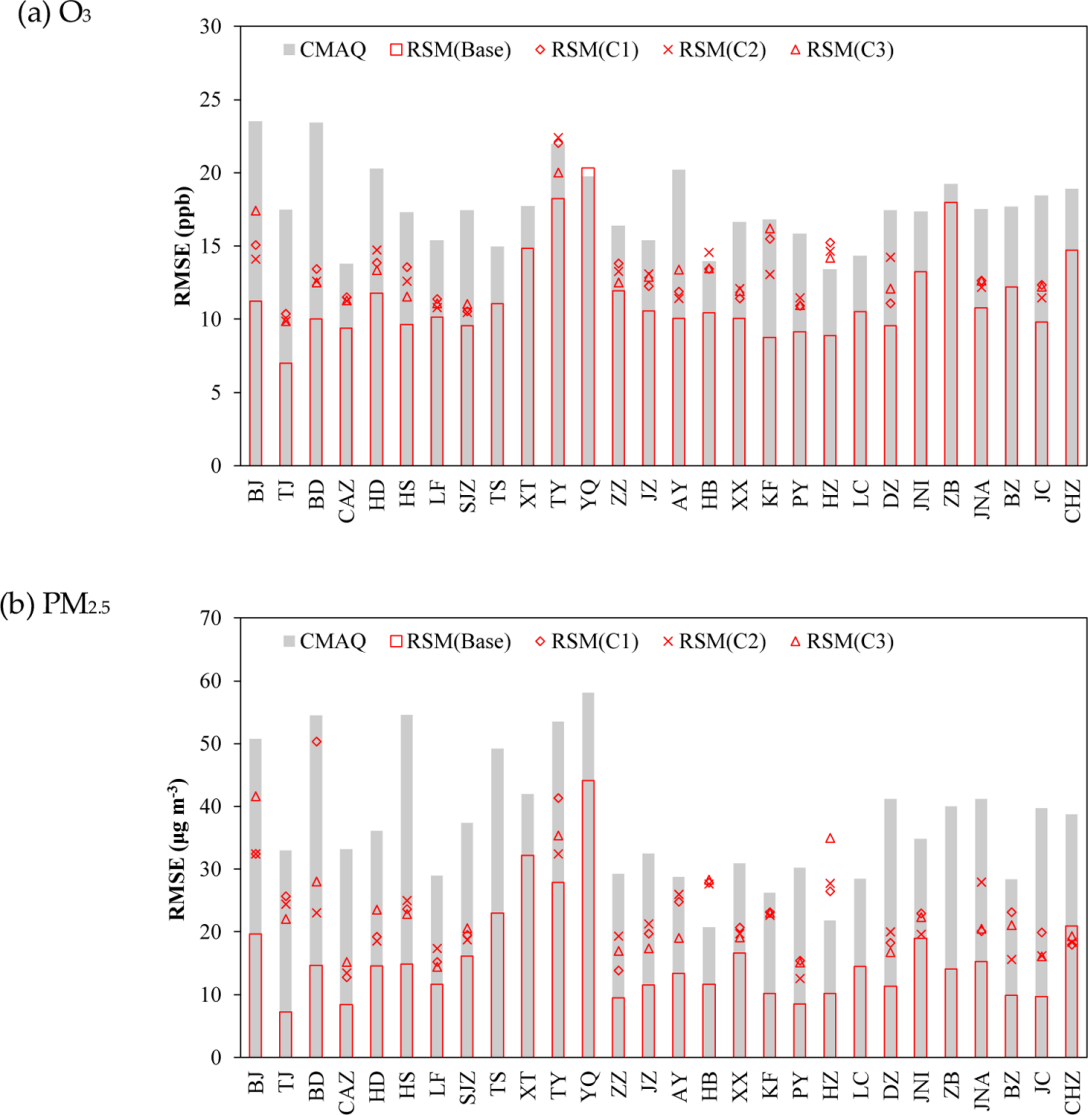
The performance of RSM-assimilation by using different number of observation sites

For traditional model-observation fusion methods, the abundance of observations has significant impacts on model performance [8]. However, in RSM-assimilation, the adjustment of emissions is done at the city-level, and therefore decreasing the number of observations within each city should have relatively less influence on performance compared to regression-based methods. To investigate the sensitivity of RSM performance to the number of sites used in assimilation, we examined performance for cases based on different numbers of observation sites, as shown in Figure 6.

In assimilation for the baseline case (red hollow bar), all observation sites were used. For the other cases (C1 to C3, red symbols), a single site in each city was used based on three random site selections. Compared to the baseline RSM, the performance does not decrease considerably for the single-site RSM-assimilation cases, C1-C3, especially for PM_2.5_. When the number of observation sites are decreased from 85 to 28, the RMSE in O_3_ predictions increases slightly (by 13%, from 11.5 to 13.3 ppb) and RMSE in PM_2.5_ increases slightly (by 42%, from 15.7 to 22.4 μg m^−3^) but still decreased by 40% from that in CMAQ (37.3 μg m^−3^) based on the average of all 28 cities. We note that the evaluation for C1-C3 was performed while withholding observations from the sites used for assimilation, thus implying that such improvement through assimilation also applies to the locations where air pollution is not monitored.

RSM-assimilation has advantages in cases where few observation data are available. However, since emissions are adjusted at the city level, the RSM-assimilation method has limited flexibility for adjusting spatial patterns of O_3_ and PM_2.5_ concentrations. The spatial distribution of emissions, which are assumed to be accurate in RSM-assimilation, might also have uncertainties that could influence the performance of assimilation in terms of representing concentration gradients. In that case, adjustment of total emissions cannot reduce the biases associated with the spatial concentration gradients, which may limit the overall performance of RSM-assimilation.

### 3.3 Implication of uncertainties in anthropogenic emissions

In addition to reducing the RMSE of model predictions, RSM-assimilation provides the emission ratios for five pollutants that are adjusted simultaneously during assimilation. The adjusted emission ratios provide information about potential uncertainties in anthropogenic emissions.

**Figure 7.**
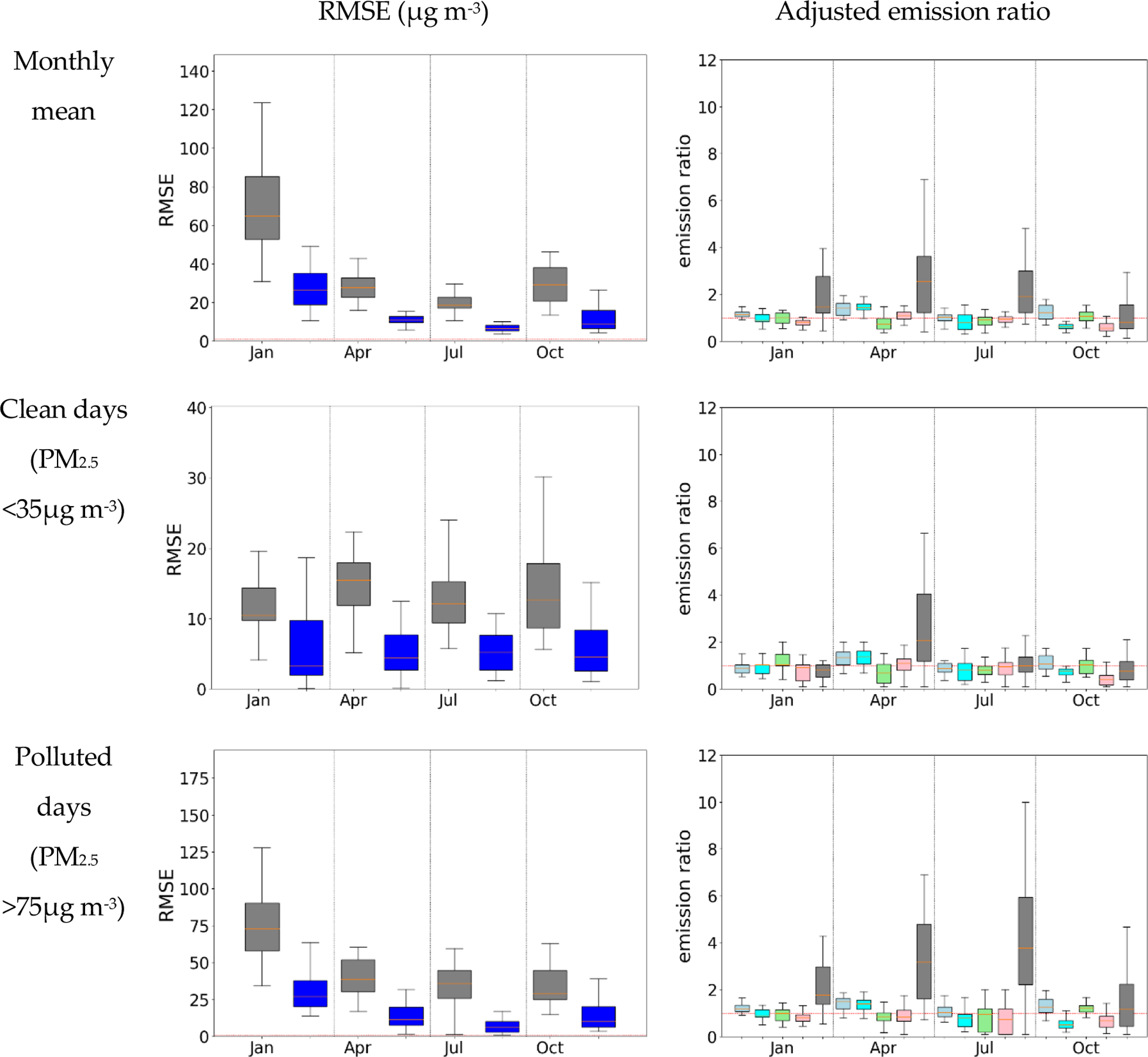
The RMSE (left: grey-CMAQ; blue-RSM) and Adjusted emission ratio for the air quality assimilation (right, baseline=1. lightblue-NO_x_; cyan-SO_2_, green-NH_3_; pink-VOC; grey-pPM_2.5_) at different polluted levels

As shown in Figure 7, for average month values, NO_x_ emissions appear to be underestimated in the baseline case based on emission adjustment ratios >1 in January, April, and October. VOC emissions appear to be overestimated (adjustment ratios <1) in January and October, and SO_2_ emissions are underestimated in April. The emission adjustment ratio for pPM_2.5_ is the largest among all pollutants, in part due to the wider range of possible changes allowed in the RSM-assimilation. The pPM_2.5_ ratio is much greater than 1 in all cases and indicates a significant underestimation, particularly in April during the dust season. The biases in assimilated concentrations generally do not reach to zero due to the limited range of emission adjustment available in RSM-assimilation. For cases that have large uncertainties in the prior emissions, the simulated concentrations cannot fully be assimilated such that predictions match the observation.

An underestimation of NO_x_ and SO_2_ emissions is evident on both clean and polluted days. On clean days, pPM_2.5_ emissions are significantly underestimated in April. On polluted days, NO_x_ emissions are underestimated during all seasons except summer. For all days, VOC emissions are overestimated in January and October, and SO_2_ emissions are underestimated in January and April but overestimated in July and October. The adjusted emission ratio for pPM_2.5_ is greater than 1 across all four months suggesting a broad underestimation of primary PM_2.5_ emissions. The uncertainties of pPM_2.5_ emissions may be due to the lack of inline dust simulation over NCP domain and underestimation of wind-blown dust emissions outside of NCP [31], as such underestimation of PM_2.5_ is more pronounced in April during the dust season. Also, the underestimation of primary organic aerosol and intermediate VOC emissions appears to have resulted in relatively larger low-biases of simulated organic aerosols than other components in April (see Figure S5). Although increasing the pPM_2.5_ emissions is an efficient way to resolve differences between modeled and observed PM_2.5_ concentrations, the emission adjustment of pPM_2.5_ also likely corrects for other model limitations. The large adjustment of pPM_2.5_ (by over two) suggests that other model uncertainties (e.g., aerosol-PBL dynamic interactions, chemical reaction rates, and potential missing chemical reaction pathways) could also play an important role in contributing to low biases. This is also suggested from the evaluation of PM_2.5_ component concentrations which imply that potential missing chemical reaction pathways for the transition of S(IV) to S(VI) [32, 33] might contribute to the low-biases of sulfate aerosols in January and July (i.e., SO_2_ concentration was overestimated but the percentage of sulfate aerosols in total PM_2.5_ was underestimated, see Figure S5). Further decoupling the influence of these processes can be done using advanced machine learning technology with the inclusion of certain feature data such as meteorological variables.

## 4. Summary and Conclusions

In this study, we developed a new assimilation method (RSM-assimilation) that uses an emission-concentration response model for assimilating PM_2.5_ and O_3_ observations simultaneously. The successful application of RSM-assimilation indicates that significant improvements in the agreement of predictions and observations can be achieved solely by adjusting the emissions. For instance, the RMSE for O_3_ and PM_2.5_ predictions decreased by about 35% and 58%, respectively, demonstrating the effectiveness of the new assimilation method based on the emission-concentration response model. We found that the RSM-assimilation has limited ability for assimilating concentrations at the edge of the control region due to the influence of emissions from surrounding regions. Compared to PM_2.5_, O_3_ concentrations are harder to assimilate with the new method due in part to the larger contributions from background and natural sources. An advantage of RSM-assimilation is that it requires very little observational data, since it uses prior knowledge of the spatial distribution of emissions. Therefore the approach is most suitable for cases where few observation are available.

The RSM-assimilation also provides useful information to improve understanding of uncertainties in the emission inventory. Based on the adjusted emission ratios, we found that the NO_x_ emissions are likely underestimated in January, April, and October; SO_2_ emissions are likely underestimated in April; NH_3_ emissions are likely overestimated in April; and VOC emissions are likely overestimated in January and October. Also, pPM_2.5_ emissions appear to be significantly underestimated in April during the dust season. Despite the success of the RSM-assimilation application, some limitations were identified that require future improvement. For example, due to the lack of NH_3_ observations, we adjusted the NH_3_ emissions simultaneously with pPM_2.5_ emissions in this study. Future work can be conducted by implementing both surface measurements and satellite retrievals (e.g., NO_2_, SO_2_, NH_3_, and HCHO) to optimize the emission accuracy across the whole domain. The inclusion of NH_3_ observations can improve the method to better constrain the adjustment for PM_2.5_. Further improvement can be also done to separate the emissions by sectors with additional correction functions such as the spatial pattern of each emission category based on observations like satellites. In addition, we assumed that the emissions changes have immediate impact on PM_2.5_ and O_3_ concentrations, but incorporating an assimilation time window into the method is necessary to account for the time needed for pollution transport and chemical formation upwind of the monitors.

## Supplementary Material

Supporting Info **Supplementary Materials:** The following are available online at www.mdpi.com/xxx/s1, Table S1: number of sites used for nudging in each city, Figure S1: Spatial distribution of five air pollutants emissions of 28 cities in NCP (unit: kt·grid^−1^·yr^−1^), Figure S2: Comparions of CMAQ-simulated, observed, and RSM-assimilated PM_2.5_ concentrations in Apr 2017, Figure S3: Comparison of observed, CMAQ-simulated and RSM-assimilated O_3_ concentration, Figure S4: Comparison of observed, CMAQ-simulated and RSM-assimilated PM_2.5_ concentration, Figure S5: Comparison of observed and simulated PM_2.5_ chemical component in a Beijing urban site (relative percentage in total PM_2.5_ mass concentration).

## Annex: abbreviations in the text

CMAQ model: Community Multiscale Air Quality model
CTM: chemical transport model
DS: Downscaler
eVNA: enhanced Voronoi Neighbor Averaging
HCHO: Formaldehyde
MEGAN: Model for Emissions of Gases and Aerosols from Nature
MEIC: Multi-resolution Emission Inventory for China
NCP: North China Plain
NH_3_: Ammonia
NMB: Normalized Mean Bias
NO_x_: nitrogen oxides
O_3_: ozone
PBL: planetary boundary layer
PM_2.5_: fine particulate matter
pPM_2.5_: primary fine particulate matter
R^2^: R-squared
RMSE: Root Mean Square Error
RSM: response surface model
SO_2_: Sulfur dioxide
VNA: Voronoi Neighbor Averaging
VOC: volatile organic compounds
WRF model: Weather Research and Forecasting model
