# Supplementary material for: Data assimilation of ambient concentrations of multiple air pollutants using an emission-concentration response modeling framework
Source: Atmosphere (Basel). Author manuscript; Available in PMC 2021 Jan 7. (PMC7787966; doi:10.3390/atmos11121289)
Supplement: Supporting Info — Supplementary Materials: The following are available online at www.mdpi.com/xxx/s1, Table S1: number of sites used for nudging in each city, Figure S1: Spatial distribution of five air pollutants emissions of 28 cities in NCP (unit: kt·grid−1·yr−1), Figure S2: Comparions of CMAQ-simulated, observed, and RSM-assimilated PM2.5 concentrations in Apr 2017, Figure S3: Comparison of observed, CMAQ-simulated and RSM-assimilated O3 concentration, Figure S4: Comparison of observed, CMAQ-simulated and RSM-assimilated PM2.5 concentration, Figure S5: Comparison of observed and simulated PM2.5 chemical component in a Beijing urban site (relative percentage in total PM2.5 mass concentration). [file NIHMS1651822-supplement-Supporting_Info.docx]

**Supplementary information for the manuscript *Data assimilation of ambient concentrations of multiple air pollutants using an emission-concentration response modeling framework***

**Table S1** number of sites used for nudging in each city

|  | BJ | TJ | BD | CAZ | HD | HS | LF | SJZ | TS | XT | TY | YQ | ZZ | JZ | AY | HB | XX | KF | PY | HZ | LC | DZ | JN | ZB | JN | BZ | JC | CHZ | TOT |
| --- | --- | --- | --- | --- | --- | --- | --- | --- | --- | --- | --- | --- | --- | --- | --- | --- | --- | --- | --- | --- | --- | --- | --- | --- | --- | --- | --- | --- | --- |
| JAN | 12 | 8 | 5 | 2 | 2 | 3 | 2 | 2 | 1 | 1 | 6 | 1 | 3 | 2 | 6 | 2 | 2 | 2 | 2 | 2 | 1 | 6 | 2 | 1 | 4 | 2 | 2 | 1 | 85 |
| APR | 12 | 11 | 5 | 2 | 4 | 3 | 2 | 2 | 1 | 1 | 6 | 1 | 3 | 2 | 4 | 3 | 2 | 2 | 2 | 2 | 1 | 6 | 2 | 1 | 4 | 2 | 2 | 2 | 90 |
| JUL | 12 | 11 | 5 | 2 | 4 | 3 | 2 | 2 | 1 | 1 | 6 | 1 | 3 | 2 | 6 | 3 | 2 | 3 | 2 | 2 | 1 | 6 | 2 | 1 | 4 | 1 | 3 | 2 | 93 |
| OCT | 3 | 9 | 5 | 2 | 4 | 3 | 2 | 2 | 1 | 1 | 6 | 1 | 3 | 2 | 6 | 3 | 2 | 2 | 2 | 2 | 1 | 6 | 1 | 1 | 4 | 1 | 3 | 2 | 80 |

| 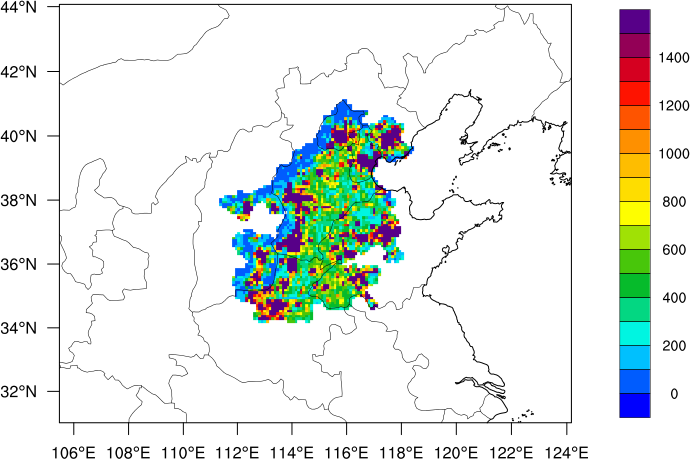 | 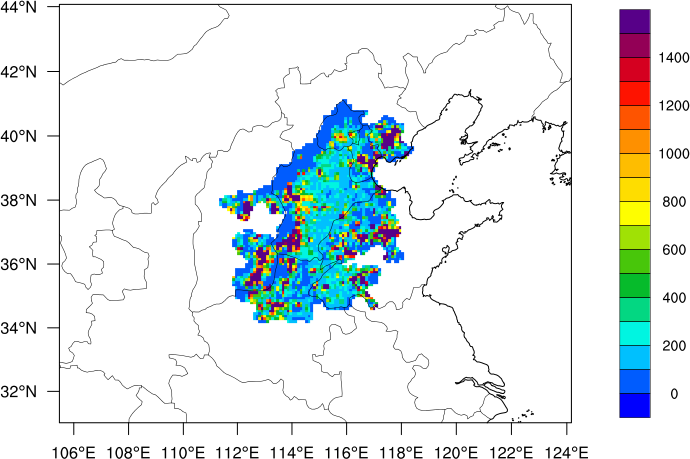 | 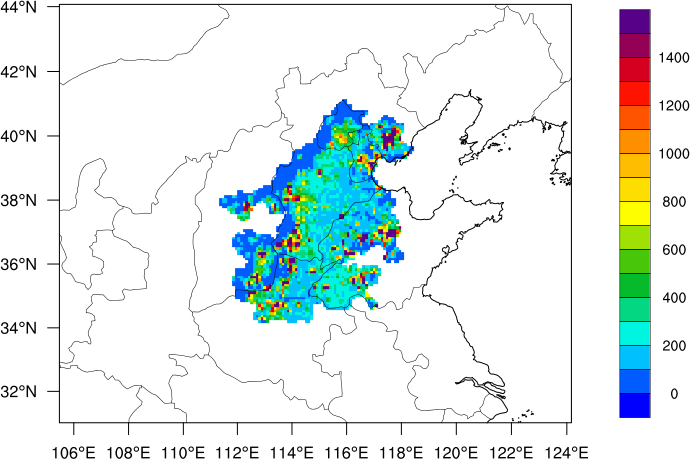 |
| --- | --- | --- |
| (a) NO_x_ | (b) SO_2_ | (c) pPM_2.5_ |
| 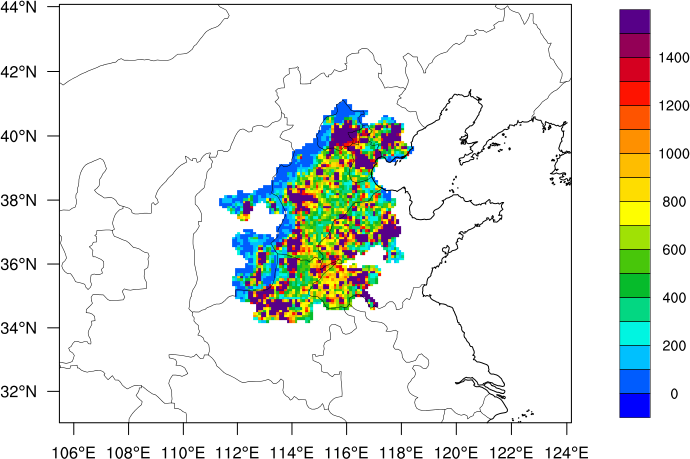 | 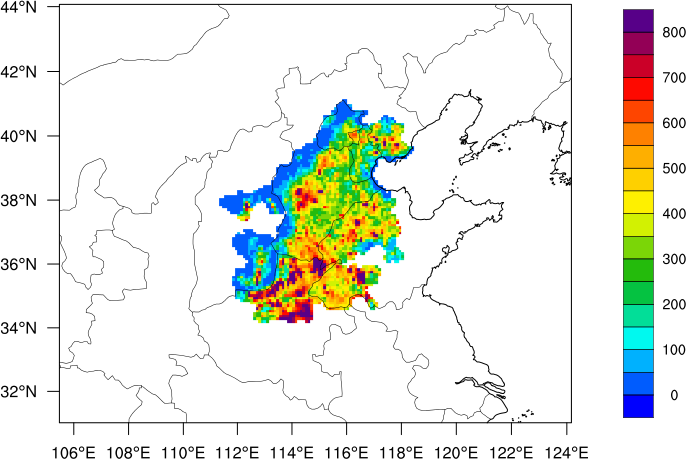 |  |
| (d) VOC | (e) NH_3_ |  |

**Figure S1** Spatial distribution of five air pollutants emissions of 28 cities in NCP (unit: kt·grid^-1^·yr^-1^)

| 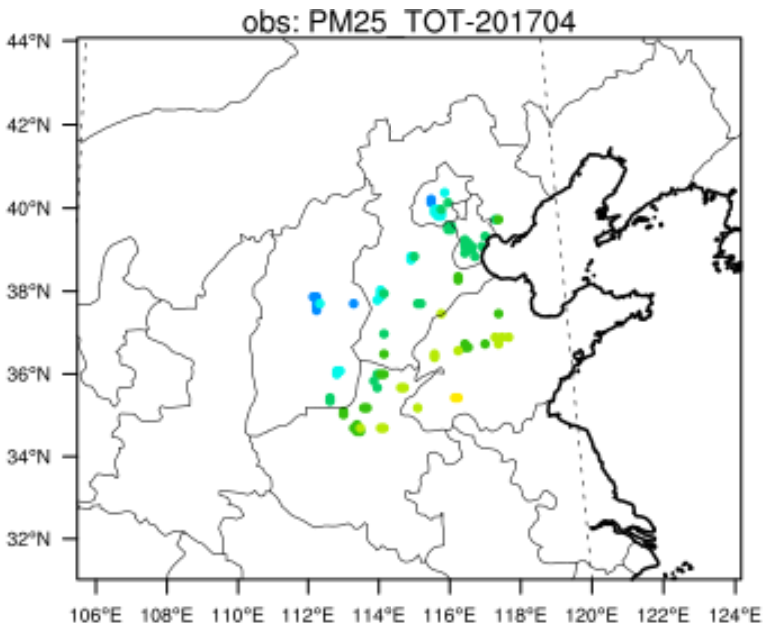 | 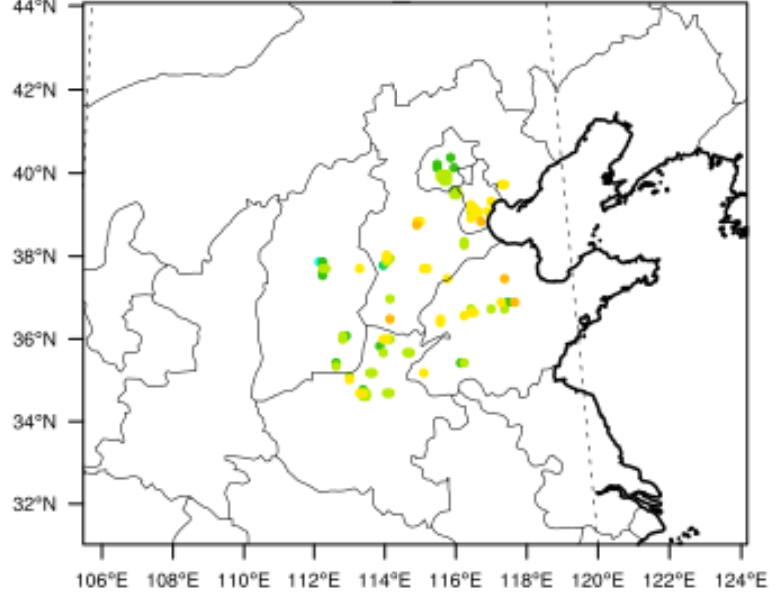 | 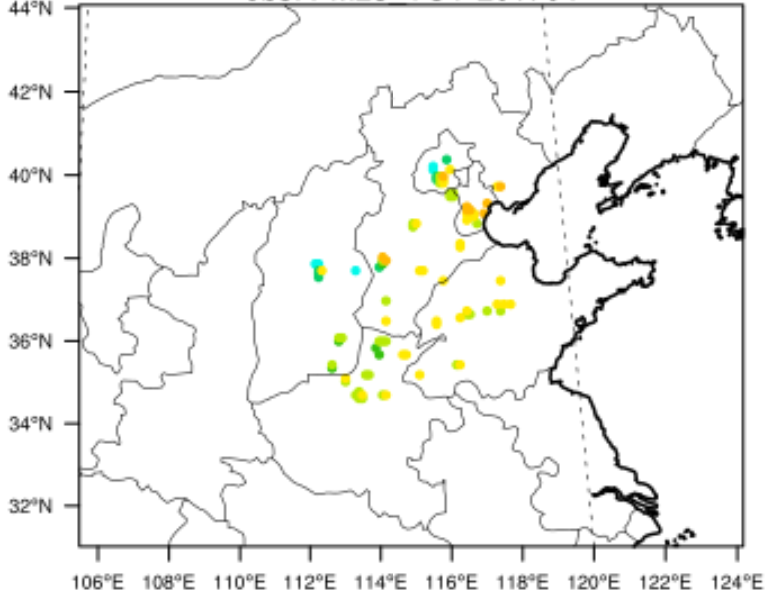 |
| --- | --- | --- |
| 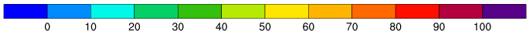 | | |

**Figure S2** Comparions of CMAQ-simulated, observed, and RSM-assimilated PM_2.5_ concentrations in Apr 2017


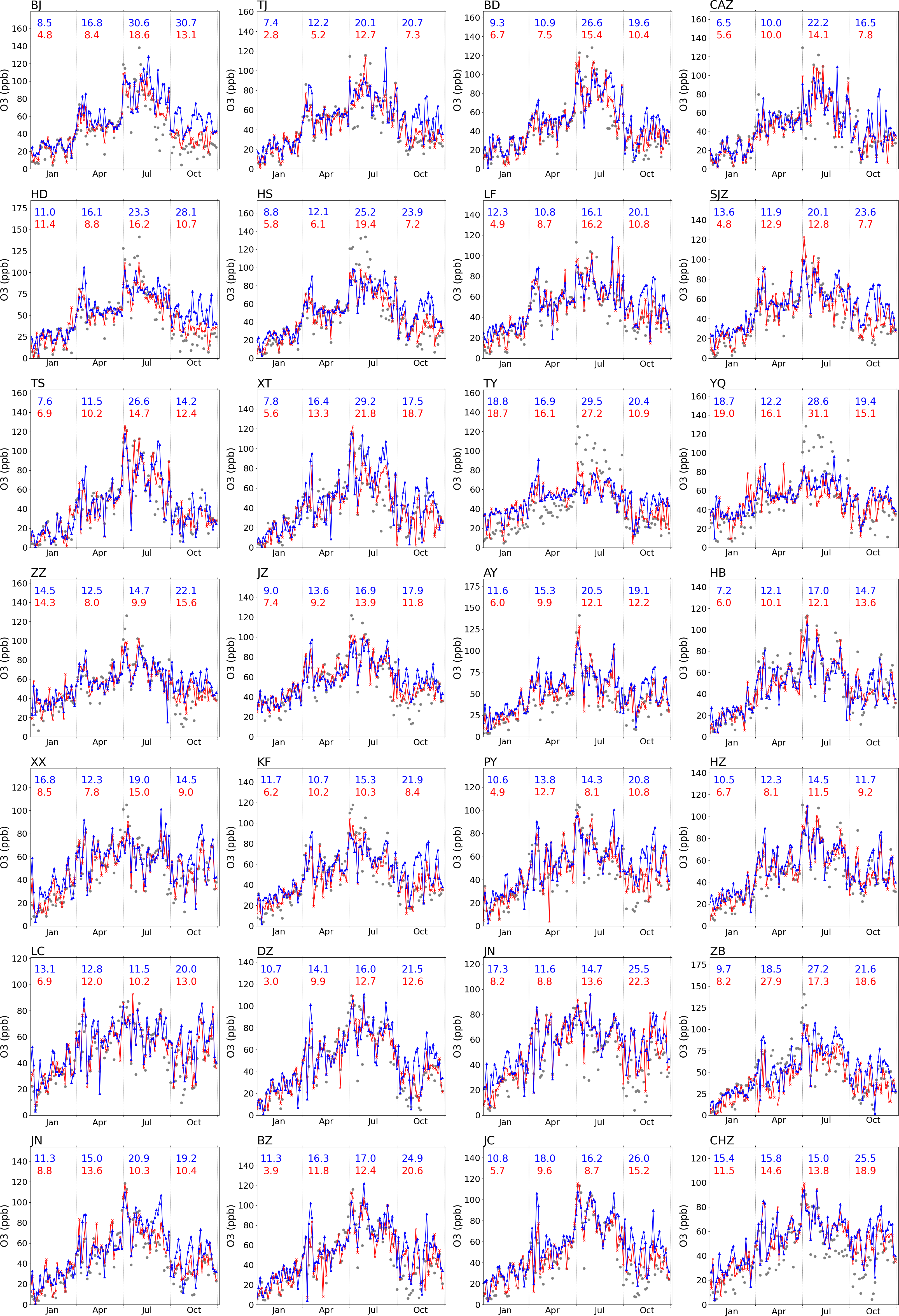


**Figure S3** Comparison of observed (grey), CMAQ-simulated (blue) and RSM-assimilated (red) O_3_ concentration (the numbers above represent RMSE in each month by cities)


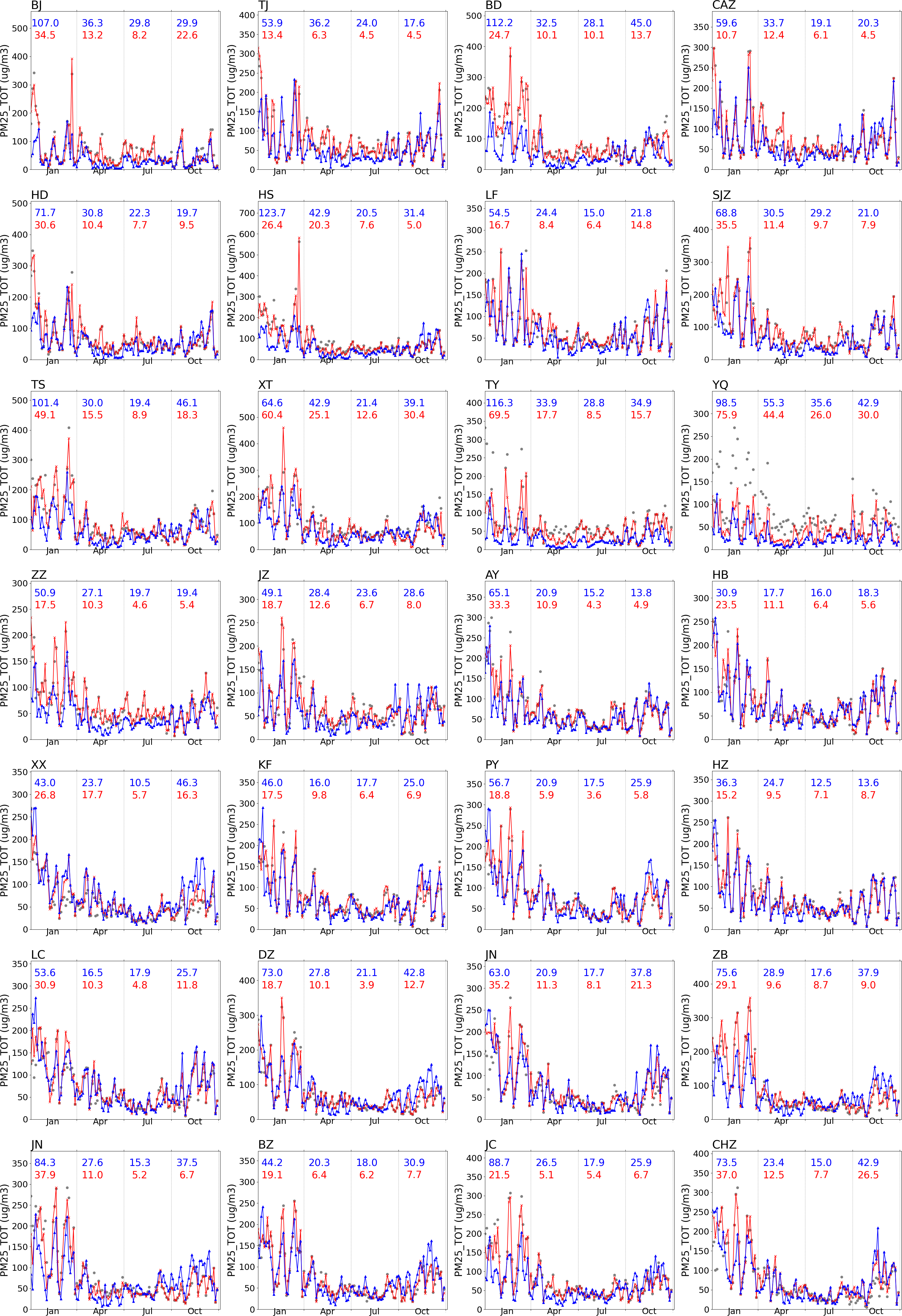


**Figure S4** Comparison of observed (grey), CMAQ-simulated (blue) and RSM-assimilated (red) PM_2.5_ concentration (the numbers above represent RMSE in each month by cities)

|  | Jan | Apr | Jul | Oct |
| --- | --- | --- | --- | --- |
| Obs |  |  |  |  |
| Sim |  |  |  |  |

**Figure S5** Comparison of observed and simulated PM_2.5_ chemical component in a Beijing urban site (relative percentage in total PM_2.5_ mass concentration)
